# Green Tea Consumption after Intense Taekwondo Training Enhances Salivary Defense Factors and Antibacterial Capacity

**DOI:** 10.1371/journal.pone.0087580

**Published:** 2014-01-30

**Authors:** Shiuan-Pey Lin, Chia-Yang Li, Katsuhiko Suzuki, Chen-Kang Chang, Kuei-Ming Chou, Shih-Hua Fang

**Affiliations:** 1 School of Pharmacy, China Medical University, Taichung, Taiwan; 2 Department of Genome Medicine, College of Medicine, Kaohsiung Medical University, Kaohsiung, Taiwan; 3 Faculty of Sport Sciences, Waseda University, Saitama, Japan; 4 Sport Science Research Center, National Taiwan University of Physical Education and Sport, Taichung, Taiwan; 5 Department of Combat Sports, National Taiwan University of Physical Education and Sport, Taichung, Taiwan; 6 Institute of Athletics, National Taiwan University of Physical Education and Sport, Taichung, Taiwan; University of Sao Paulo, Brazil

## Abstract

The aim of this study was to investigate the short-term effects of green tea consumption on selected salivary defense proteins, antibacterial capacity and anti-oxidation activity in taekwondo (TKD) athletes, following intensive training. Twenty-two TKD athletes performed a 2-hr TKD training session. After training, participants ingested green tea (T, caffeine 6 mg/kg and catechins 22 mg/kg) or an equal volume of water (W). Saliva samples were collected at three time points: before training (BT-T; BT-W), immediately after training (AT-T; AT-W), and 30 min after drinking green tea or water (Rec-T; Rec-W). Salivary total protein, immunoglobulin A (SIgA), lactoferrin, α-amylase activity, free radical scavenger activity (FRSA) and antibacterial capacity were measured. Salivary total protein, lactoferrin, SIgA concentrations and α-amylase activity increased significantly immediately after intensive TKD training. After tea drinking and 30 min rest, α-amylase activity and the ratio of α-amylase to total protein were significantly higher than before and after training. In addition, salivary antibacterial capacity was not affected by intense training, but green tea consumption after training enhanced salivary antibacterial capacity. Additionally, we observed that salivary FRSA was markedly suppressed immediately after training and quickly returned to pre-exercise values, regardless of which fluid was consumed. Our results show that green tea consumption significantly enhances the activity of α-amylase and salivary antibacterial capacity.

## Introduction

Many factors are present in mucosal secretions, including immunoglobulins (Igs), α-amylase and anti-microbial peptides (AMPs) [1]–[3], which serve as the first line of defense against microbial infection. Salivary IgA (SIgA) contributes to mucosal immunity by preventing adherence of microbes to the mucosal surface [4]. Amylase functions as an antibacterial protein inhibiting bacterial growth and colonization in the oral cavity [5], [6]. Lactoferrin, one of the most abundant salivary AMPs, exerts an antibacterial effect by sequestering iron, an essential nutrient for bacterial growth, as well as directly interacting with, and damaging, bacterial membranes [7], [8]. Additionally, saliva contains various kinds of anti-oxidative systems that guard against oxidative damage induced by free radical-mediated oxidative stress [9]. Salivary total antioxidant capacity can be evaluated by measuring the free radical scavenging activity (FRSA) [10]. However, salivary exertion of these defense factors can be affected by high intensity exercise [11]. Prolonged, strenuous exercise has been implicated in immunosuppression, induction of inflammatory response and increased production of free radicals [12]–[14]. Therefore, development of nutritional strategies to alleviate the negative effects of intensive exercise would seem particularly desirable.

Green tea is a non-fermented/oxidized tea that contains various polyphenolic flavonoids, including epicatechin, epicatechin gallate, epigallocatechin and epigallocatechin gallate [15]. Consumption of green tea has been reported as beneficial for health given its anti-angiogenic, anti-carcinogenic and anti-diabetic activities [16]–[18]. It has been suggested that the pharmacological properties of green tea may be mediated, at least partially, by its potent anti-oxidative activity, anti-inflammatory and immunomodulatory effects [19]–[21]. Thus, it is reasonable to assume that the detrimental effects caused by intensive exercise may be alleviated by consumption of green tea. In recent years, there is a growing interest to understand the potential beneficial role of green tea in exercise performance and recovery from high intensity exercise. Studies have revealed that habitual consumption of green tea improves endurance capacity and exercise performance in both mice and humans [22]–[25]. Recently, it was demonstrated that oral supplementation of theanine, abundant in green tea, and cystine can significantly attenuate exercise-induced peripheral neutrophilia and lymphopenia [26]. Although previous research has indicated that consumption of green tea can effectively alleviate some of the negative effects caused by high-intensity exercise, its effects on salivary defense proteins, antibacterial capacity and total antioxidant activity are still unclear.

Taekwondo (TKD) is a high speed, high tension, full-contact combat sport, and the training program for TKD athletes includes a series of intensified, vigorous physical exercises. Our previous results suggested that the cumulative effects of prolonged, strenuous TKD training in combination with rapid weight loss can significantly suppress the mucosal immunity of male and female TKD athletes [27], [28]. However, short-term effects of high intensity TKD training on individual mucosa immunity following green tea consumption are still poorly understood. Therefore, the aim of this study was to investigate the effect of acute green tea consumption following intensive TKD training on salivary defense factors and antibacterial capacity of male and female athletes.

## Materials and Methods

### Participants

Twenty-two TKD athletes (13 males and 9 females) from the National Taiwan University of Physical Education and Sport TKD team volunteered to participate in this study. Athletes who needed to take any medication during this study were excluded. This study protocol was approved by the Human Ethics Committee of the National Taiwan University of Physical Education and Sport before the start of this study. Written informed consent was obtained from each participant after detailed explanation of the study.

### Determination of physical characteristics

Body weight, body fat and percent body fat were measured by an eight-electrode bioimpedance analyzer (InBody 3.0, Biospace, Seoul, Korea). Height was measured using a stadiometer (Holtain, UK) to the nearest 0.1 cm. Body mass index (BMI) was calculated as body weight (kg) divided by the square of height (m). Physical characteristics of the participants at the beginning of the study are summarized in Table 1.

**Table 1:** Participant characteristics.

Group	Male	Female
Number of athletes	13	9
Age (years)	20.5±1.2	19.9±1.5
Height (cm)	175.7±6.6	166.3±6.4
Body weight (kg)	68.9±9.9	58.4±5.6
Body fat (kg)	10.7±5.5	12.7±3.4
Body fat (%)	15.1±4.9	21.7±4.8
BMI (kg/m^2^)	22.2±2.2	21.1±1.7

Values are mean ± SD.

BMI, body mass index.

### Study design

Each participant performed two identical 2-hr TKD training sessions at the same time of day (15:00), separated by one week. The intensity of each training session was 80–85% of predicted maximum heart rate. Approximately 1-hr of the training session was devoted to technique training and 1-hr was physical training. The technique training component of the session included basic techniques, simulated fighting techniques and simulated matches. The physical training component of the session included aerobic activities. Participants were allowed to drink water *ad libitum* during the training session. Immediately after training, saliva was collected and then all TKD athletes were given a single oral dose of green tea of 9 ml per kg of body mass (containing caffeine 6 mg/kg and catechins 22 mg/kg) on the first training session and an equal volume of distilled water on the second training session one week later.

### Preparation of green tea

Dried non-fermented green tea leaves (Pi-Lo-Chun green tea) were purchased from Ten Ren Tea Company (Taipei, Taiwan). Extraction was carried out by soaking 20 g of green tea leaves in 600 ml of distilled water at 25°C for 24 h. These infusions were then filtered through a tea strainer.

### Determination of caffeine and catechins compositions of green tea

The quantification of caffeine and catechins compositions followed Yang's method with minor modification [29]. In brief, liquid was passed through a 0.45 µm filter and then 20 µl of sample was injected into a RP-18e column (LiChrospher, 250×4.0 mm, Merck, Darmstadt, Germany). Gradient elution was performed at a flow rate of 1 ml/min. The solvent system used was a gradient of solvent A (acetonitrile) and solvent B (0.9% acetic acid). The gradient was as follows: 0–5 min, 5% A; 6–26 min, linear gradient 5–13% A; within 27 min, linear gradient 13–27% A; 28–39 min, 27% A; within 40 min, linear gradient 27–100% A; 41–50 min, 100% A; 51–60 min, 5% A. Identification of caffeine and catechins compositions in tea infusions was carried out by comparing the retention time with caffeine and catechins standards. Quantitative analyses for caffeine and catechins compositions in green tea infusions were performed in triplicate.

### Saliva collection

Unstimulated whole saliva was collected as described previously [27]. All participants were seated and asked to thoroughly rinse their mouth with 30 ml of sterile distilled water before sample collection. Participants remained seated for 10 minutes and until all the saliva samples were then collected into sterile plastic containers. Two ml of saliva samples were collected at three time points: before training (BT), immediately after training (AT), and 30 min after drinking water or green tea (Rec). The saliva samples were stored immediately at −80°C until assay.

### Antibacterial assay

*E. coli* were cultured in Luria-Bertani (LB) broth in a shaking incubator set at 37°C, 130 rpm until OD 600 nm reached a value of approximately 0.6 to 0.7. The bacteria suspension was diluted 10^6^ times in LB broth. Subsequently, 400 µl of the diluted *E. coli* culture were mixed with 300 µl of freshly thawed saliva samples or PBS as a control, and the mixtures were placed in a shaking incubator set at 37°C, 130 rpm. After 1 hour, 100 µl of the cultures were spread plated onto 37°C -pre-warmed LB agar plates and incubated in a 37°C incubator overnight. The colony-forming units (CFU) were counted and the antibacterial activity of saliva was presented as: (%)  =  [(CFU of control−CFU of experiment)/CFU of control] X 100. All assays were performed in triplicate.

### Saliva assays

Levels of total protein, SIgA, lactoferrin and FRSA of each saliva sample were measured as described in our previous study [27]. Total protein concentrations were determined using the Bio-RAD protein assay kit (Bio-RAD, Hercules, CA, USA). SIgA and lactoferrin concentrations were measured using enzyme-linked immunosorbent assays (ELISA, Calbiochem, Darmstadt, Germany) following the manufacturer's instructions. Salivary FRSA was measured by mixing 20 µl of saliva to 2,2-diphenyl-1-picrylhydrazyl (DPPH) solution (0.2 mM dissolved in 40% ethanol), and then measuring the absorbance at 540 nm. FRSA was assayed according to the former method [30] and presented as the percentage of decreased DPPH compared with a control experiment without the addition of saliva and was calculated by the following formula: FRSA (%)  =  ((Ac - As) ÷ Ac) ×100, where Ac and As represent the absorbance with the addition of PBS and saliva, respectively. The α-amylase activity was determined using a kinetic reaction assay kit (Salimetrics LLC, State College, PA, USA) according to the manufacturer's instruction. To prevent any change induced by hydration in saliva, the changes of salivary proteins normalized with TP also be monitored. All samples were measured in triplicate. The intra-assay coefficient of variation (CV) for the measurements of SIgA, lactoferrin and α-amylase activity was 3, 3 and 4%, respectively.

### Statistical analyses

All data are expressed as mean±SD. Statistical comparisons between different time points were analyzed using Wilks' lambda test of multivariate analysis of variance (MANOVA). Significant difference was set at ^#^ *P*<0.05 and ^##^ *P*<0.01. Statistical comparisons between tea group and water group were analyzed using paired t-test. Significant difference was set at ^*^ *P*<0.05.

## Results

### Anthropometric differences between male and female participants

Although male TKD athletes were significantly taller, heavier and had a lower percent body fat than the female TKD athletes, BMI value was similar among all participants (Table 1).

### The content of caffeine and catechins compositions in the green tea

The concentrations of the caffeine and catechins compositions in the cold-prepared green tea were caffeine, 688.3 µg/ml; (−)-epigallocatechin (EGC), 1155.3 µg/ml; (+)-catechin (C), 90.1 µg/ml; (−)-epicatechin (EC), 228.6 µg/ml; (−)-epigallocatechin gallate (EGCG), 1030.3 µg/ml; and (−)-epicatechin gallate (ECG), 137.4 µg/ml.

### Salivary antibacterial capacity

**Figure 1:**
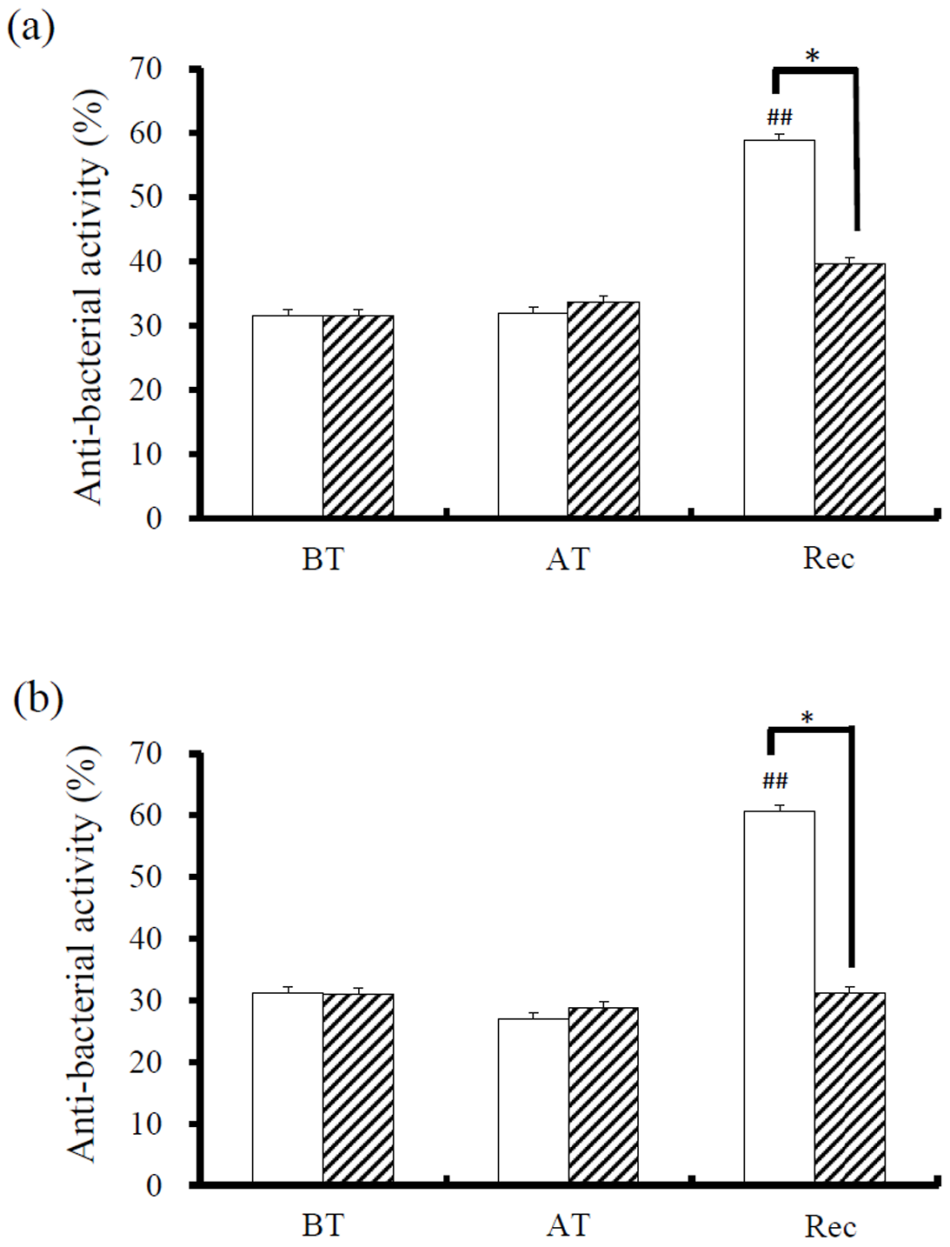
Antibacterial activity of saliva collected from (a) male and (b) female athletes at various time points. The antibacterial activity of saliva was presented as: (%)  =  [(CFU of control−CFU of experiment)/CFU of control] X 100. All assays were performed in triplicate. Significant difference between ingestion of water (white bar) and green tea (slashed bar) at each sampling time was set at *P<0.05. Significant difference between each sampling time and the initial condition (BT) was set at *^#^P*<0.05 and ^##^ *P*<0.01. CFU, colony-forming units; BT, before training; AT, after training; Rec, 30 min of recovery after ingestion of water or tea.

Salivary antibacterial capacity of male and female participants was not significantly different before and after intensive TKD training (Fig. 1; BT vs. AT). However, consuming green tea significantly stimulated the salivary antibacterial capacity of participants after 30 min rest (Fig. 1; BT vs. Rec). A similar response pattern was observed in male and female athletes (Fig. 1a and Fig. 1b)

### Salivary total protein concentrations

Immediately after TKD training, salivary total protein concentrations of male and female athletes were significantly increased (Table 2, BT vs. AT). Total protein concentrations returned to pre-exercise values 30 minutes following ingestion of water (BT-W vs. Rec-W), but not after ingestion of green tea (BT-T vs. Rec-T).

**Table 2:** Concentrations of salivary total protein, lactoferrin, SIgA and α-amylase activity.

	Condition	Total protein	α-Amylase	Lactoferrin	SIgA	α-Amylase/TP	Lactoferrin/TP	SIgA/TP
		(µg/ml)	(U/ml)	(ng/ml)	(µg/ml)	(U/ml)	(ng/ml)	(µg/ml)
Male								
	BT-T	883.5±368.1	27.3±12.8	2234.7±620.0	213.1±42.5	32.9±12.4	3324.1±961.2	241.2±66.6
	AT-T	1372.5±648.0#	43.3±23.1^##^	3057.5±620.8#	277.0±94.8#	34.0±14.5	2726.3±675.2	222.1±47.4
	Rec-T	1390.7±615.5*,#	63.2±34.5*,##	2506.3±302.4	268.4±91.3#	49.7±16.1*,#	2015.4±366.9*,#	218.5±77.8*,#
	BT-W	879.3±374.3	26.2±15.7	2343.2±620.5	203.5±65.4	29.4±14.3	3305.8±983.2	253.1±66.7
	AT-W	1371.3±649.5#	41.7±22.3#	3096.0±597.4#	282.5±92.9#	35.6±13.1	2641.1±742.8	222.2±47.3
	Rec-W	825.5±275.3	29.1±27.5	2634.0±328.5	223.7±65.6	31.2±17.4	3187.6±885.0	255.8±85.8
Female								
	BT-T	804.4±300.4	24.6±10.1	2552.0±207.5	147.6±76.0	26.7±9.0	3677.1±1478.9	183.5±58.3
	AT-T	1164.5±498.1#	42.2±20.4^##^	2780.3±157.7#	198.0±53.9#	30.6±14.1	2864.0±1011.3	166.0±37.7
	Rec-T	1203.0±310.4*,#	57.9±27.8*,##	2511.2±245.0	162.7±62.1#	48.8±15.4*,#	2257.7±477.2*,#	123.5±27.6*,#
	BT-W	796.0±311.7	22.2±11.7	2543.9±226.1	152.1±61.3	26.3±9.6	3657.3±998.0	184.3±58.3
	AT-W	1147.7±503.7#	39.8±21.8#	2807.2±146.6#	192.5±63.4#	27.4±14.3	2925.1±973.5	175.1±35.6
	Rec-W	837.2±286.5	24.8±19.3	2347.4±143.9	149.2±51.7	29.7±15.5	3101.7±935.2	178.7±30.6

Values are mean ± SD

SIgA, salivary immunoglobulin A; BT, before training; AT, after training; Rec-T, 30 min of recovery following ingestion of green tea;

Rec-W, 30 min of recovery following ingestion of water.

* *P*<0.05; **P<0.01, significantly different from water (W) consumption at the same time point.

# *P*<0.05; ^##^ *P*<0.01, significantly different from before training (BT) in the same consumption.

### Salivary defense factors

As shown in Table 2, α-amylase activity increased significantly immediately after training (BT vs. AT), and green tea ingestion further stimulated α-amylase activity after 30 min rest (BT-T vs. Rec-T). In addition, the ratio of α-amylase activity to total protein was significantly higher following 30 min rest (Rec-T) than before and after exercise (BT-T and AT-T) with green tea consumption. Absolute concentrations of lactoferrin increased immediately after training (BT vs. AT), and returned to pre-exercise levels after 30 min of rest (BT-T vs. Rec-T and BT-W vs. Rec-W). Concentrations of SIgA increased significantly immediately after training (BT vs. AT) and stayed high after green tea consumption and rest (Rec-T). However, the ratio of lactoferrin or SIgA concentrations to total protein was significantly lower than before and after exercise with green tea supplement.

### FRSA

**Figure 2:**
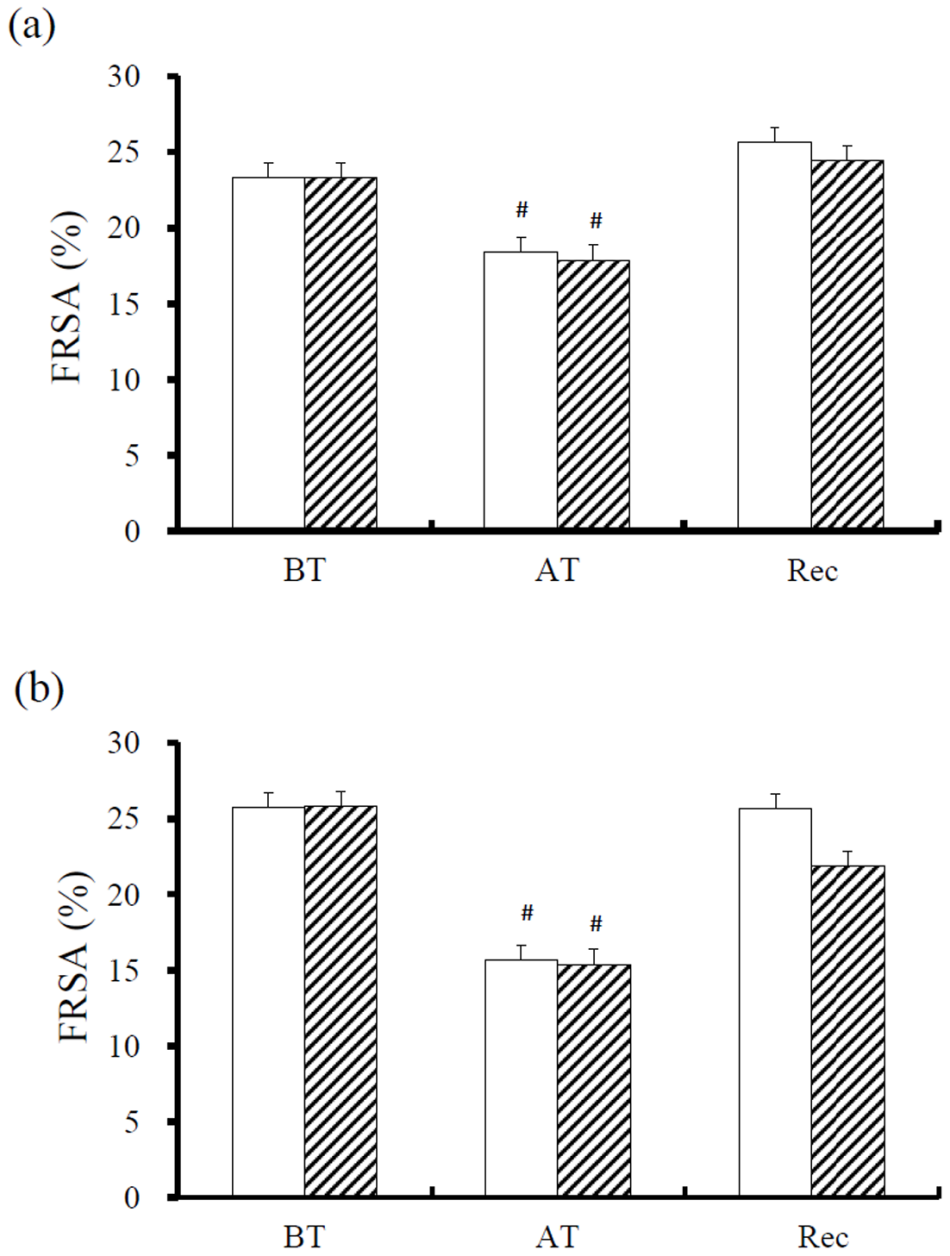
The free radical scavenger activity (FRSA) of saliva collected from (a) male and (b) female athletes at various time points. FRSA (%)  =  ((Ac - As) ÷ Ac) ×100, whereas Ac and As represents the absorbance with the addition of PBS and saliva samples from either ingestion of water (white bar) or green tea (slashed bar), respectively. Significant difference between each sampling time and the initial condition (BT) was set at ^#^ *P*<0.05. BT, before training; AT, after training; Rec, 30 min of recovery after ingestion of water or tea.

Results show that FRSA levels in male and female athletes were significantly decreased immediately after TKD training (Fig. 2; BT vs. AT). However, FRSA levels returned to pre-exercise values 30 min after training regardless of which fluid was consumed (BT vs. Rec).

## Discussion

The present study reveals that intensive TKD training increases salivary concentrations of total protein, SIgA, lactoferrin and α-amylase activities. Additionally, green tea consumption following intensive training exerts short-term effects on salivary antibacterial capacity, α-amylase activities and total protein concentrations. In this study, no significant differences between male and female athletes were observed regarding the responses of the above salivary factors to TKD training and/or green tea consumption.

Intensive TKD training did not affect the antibacterial capacity of participants. Our results are similar to those of a recent study by Davison et al. [30] who reported that although salivary defense factors were increased following prolonged exercise, salivary antibacterial capacity was not changed. While actue exercise did not affect antibacterial capacity green tea consumption significantly elevated antibacterial capacity compared to water. In this study all the athletes ingested fresh green tea at the same training session to ensure the green tea composition was uniform as different preparations may differ in caffeine and catechins content and for this reason a possible order effect cannot be ruled out. However, green tea and its major components have been previously shown to exhibit antibacterial effects [31]. There were no detectable levels of tea catechins in collected saliva (data not shown) and therefore, the increased salivary antibacterial capacity is most probably due to salivary defense factors affected by green tea consumption. Prolonged, intense exercise may alter salivary total protein, lactoferrin, SIgA and α-amylase activity, but not salivary antibacterial capacity suggesting that there may exist a complex interaction between salivary defense factors.

In addition, our results show that α-amylase activity was stimulated by TKD training and further increased 30 minutes after the ingestion of green tea. Previous studies have reported that α-amylase activity was stimulated following psychological stress or physical exercise [32], [33]. Besides starch hydrolysis activity, α-amylase has been shown to function as an antibacterial protein by inhibiting bacterial growth and colonization in the oral cavity [5], [6]. Therefore, we suggest that the increased salivary antibacterial capacity after green tea consumption could be at least partially explained by the elevated α-amylase activity. However, complex interactions between host defense factors make it difficult to evaluate the influence of individual components to host antibacterial capacity [34] and as such. Therefore, further experiments are required to elucidate the role of α-amylase in salivary antibacterial capacity.

Total protein levels increased significantly immediately after TKD training while concentrations returned to pre-exercise levels 30 minutes following water ingestion. The immediate post-exercise increase in total protein concentration is most likely attributable to acute dehydration induced by intense exercise [35]. While protein levels were reduced to pre-exercise levels following the ingestion of water post-exercise, total protein concentrations remained relatively high 30 min after consumption of green tea. However, it is well known that α-amylase is one of the most abundant proteins in saliva [36]. Our results showed that levels of α-amylase were markedly stimulated by consumption of green tea. Therefore, elevated total protein concentration may be due to the marked increases of α-amylase stimulated by green tea ingestion.

Levels of SIgA and lactoferrin were elevated by TKD training and returned to pre-exercise levels after 30 min of rest, regardless of which fluid was consumed. Although absolute concentrations of SIgA and lactoferrin were increased after intense exercise, the ratio of SIgA or lactoferrin to total protein was not significantly changed suggesting that changes in absolute concentration were attributable to acute dehydration caused by intense exercise [37]. In addition, our results indicate that the changes in lactoferrin and SIgA concentrations were not correlated with salivary anti-*E. coli* capacity. Therefore, these salivary AMPs may exert different antibacterial capacities against different strains of pathogens. The basal levels of SIgA detected in this study were similar to those of basketball players [12] and TKD athletes without performing rapid weight reduction [28]. Besides the differences caused by experimental design, the basal levels of SIgA and lactoferrin measured in this study were modestly different from those of our previous studies [12], [27], [28]. This may be accounted for by the individual variances between different participants.

Studies investigating the antioxidant activity of green tea in response to exercise have typically had participants ingest green tea prior to exercise [22], [38]. In this study, we measured FRSA to investigate the short-term effect of green tea on the recovery of exercise-induced changes in antioxidant activity. Our results indicated that total salivary antioxidant activity was significantly decreased immediately following intense TKD training and returned to pre-exercise levels after 30 min of rest. Ingestion of green tea did not significantly affect total salivary antioxidant activity. Our previous study found no accumulative effects were observed in total salivary antioxidant activity on elite TKD athletes during a period of intense training and competition if saliva samples were collected after at least 12 hour of rest [27]. In this study, we have demonstrated that total salivary antioxidant activity was acutely reduced by intense exercise and then returned to its pre-exercise level. Atsumi et al. reported that immediately following moderate exercises, total salivary antioxidant activity was significantly decreased [10]. Therefore, the transitory decrease in total salivary antioxidant activity observed here may be due to the relatively higher exercise intensity used in this study and/or more intense psychological stress. Detailed mechanisms underlying the effects of green tea ingestion on total salivary antioxidant activity are still unclear and require further investigation.

## Conclusions

Our experiment demonstrated that salivary antibacterial capacity during the post-exercise recovery period was significantly enhanced by ingestion of green tea. This enhanced antibacterial capacity might be partially mediated by increased α-amylase activity. Therefore, our results suggest that green tea consumption exerts beneficial effects on athletes following intense exercise by enhancing salivary defense against microbial pathogens.
